# Imposition in the Sale of Medicine

**Published:** 1849

**Authors:** 


### ARTICLE IV.

### IMPOSITION IN THE SALE OF MEDICINE.

We have received, through the politeness of Mr. Crawford, who reported it, a copy of a Bill introduced before the Legislature of Wisconsin, for the prevention of imposition in the sale of medicine.

On reading the first three sections of the Bill, which require all compound medicines, vended in the State, to be accompanied by a label setting forth in the English language each ingredient, and its proportion, under a penalty of five dollars for each offence, with twice the amount asked for the article added, we thought our friends were taking a step in advance; but when Section 4th exempted all patent medicines from the provisions and penalties of the bill, we exclaimed: surely our friends are throwing away their efforts!

They give to the patented nostrum the privilege of secrecy, while the physician's prescription made by him from actual examination, if sent to the apothecary, must be labeled *in English* as above specified. In other words, the patent nostrum designed to be used by the patient, by guess, so far as its applicablility is concerned has full privilege without restraint: while each prescription of the physician, made out for the special case, and of the fitness of which, he is alone to judge, must be set forth on a label in English.

We hope the bill was amended before it passed, or the title changed to set forth its true bearing, as follows: A Bill to secure exclusive privileges to the venders of patent medicines. E.

